# Clinical Outcomes of Ustekinumab in Inflammatory Bowel Disease

**DOI:** 10.7759/cureus.46833

**Published:** 2023-10-11

**Authors:** Sirisha K Gara, Prathima Guntipalli, Sima Marzban, Muhammad Taqi, Vinayak Aryal, Qurat ul ain Khan, Shahtaj A Shah, Hanieh Akbariromani, Darren Salinger, Miguel Diaz-Miret

**Affiliations:** 1 Richard M. Fairbanks School of Public Health, Indiana University, Indianapolis, USA; 2 Geriatric Research Education and Clinical Center, Veterans Affairs Palo Alto Health Care System, Palo Alto, USA; 3 Research and Academic Affairs, Larkin Community Hospital, South Miami, USA; 4 Orthopedic Surgery, Mayo Hospital, King Edward Medical University (KEMU), Lahore, PAK; 5 Pathology and Laboratory Medicine, Nepal Cancer Hospital and Research Center, Lalitpur, NPL; 6 Pathology and Laboratory Medicine, MetroHealth System/Case Western Reserve University, Cleveland, USA; 7 Internal Medicine, Services Institute of Medical Sciences, Lahore, PAK; 8 Clinical Research, Surgical ICU Research Center, Brigham and Women's Hospital, Boston, USA; 9 Medicine, Islamic Azad University, Tehran, IRN; 10 Family Medicine, Larkin Community Hospital Palm Springs Campus, Hialeah, USA

**Keywords:** interleukin-23 and ulcerative colitis, interleukin-12, interleukin-12/23 monoclonal antibody and ibd, inflammatory bowel disease, crohn's disease, ulcerative colitis, ibd, ustekinumab

## Abstract

Inflammatory bowel diseases including Crohn's disease (CD) and ulcerative colitis (UC) are characterized by abdominal pain, diarrhea, blood in stools, weight loss, and fatigue. It presents in patients with varying severity from mild to severe depending on the inflammation. Detailed analysis and guidelines are required for the safe usage of biological therapies in the treatment of inflammatory bowel diseases as surgery is reserved for more complex cases. There is also geographical variation in inflammatory bowel disease (IBD) incidence and prevalence based on environmental and climate changes, and socio-demographics. Studies also show that there is more hospitalization and reduced health-related quality of life in IBD patients when compared to normal people. We conducted an extensive literature database search for articles with keywords within the last 10 years on adults >18 years of age with IBD and its treatment, especially with ustekinumab. Ustekinumab is a human immunoglobulin G1 (IgG1) kappa monoclonal antibody, that blocks IL-12 and IL-23 and was approved by the FDA for the treatment of moderate to severe IBD, especially in patients who are intolerant to immunomodulators or corticosteroids treatment. There are several retrospective studies that show the effectiveness of ustekinumab dosage escalation every four weeks in IBD patients. This escalation of dose not only improved the clinical outcome but also reduced the worsening of the disease. Previous studies also show the importance of considering dosage escalation before switching biological agents in the IBD treatment. Ustekinumab has also demonstrated both efficacy and safety in the induction and maintenance of the treatment of this disease. There are certain challenges and opportunities associated with ustekinumab usage in IBD patients that require further research. Ustekinumab seems to be more cost-effective in the tumor necrosis factor (TNF)-alpha-inhibitor failure population when compared to previously used biological treatment regimes.

## Introduction and background

Crohn's disease (CD) and ulcerative colitis (UC) are both inflammatory bowel diseases (IBDs), with CD characterized by transmural segmental gastrointestinal tract inflammation. The CD involves chronic inflammation resulting in abdominal pain, gastrointestinal bleeding, chronic diarrhea, and complications, such as strictures and fistulas [1]. Ulcerative colitis (UC), despite the increasing use of immunosuppressants, immunomodulators, and biologics, leads to debilitating effects on patients. Although biologics, such as anti-tumor necrosis factor (TNF) are used in UC, there are still limitations. In 2016, neither pre-approval trials nor clinical observation data on the use of ustekinumab in UC were available. Several studies showed no association with cancer after ustekinumab treatment [2]. The monoclonal antibodies ustekinumab (CNTO 1275) and briakinumab (ABT874), which target the p40 subunit of interleukin 12 and interleukin 23 (IL12/23p40), have been approved for the treatment of Crohn's disease. Inhibition of these cytokines is beneficial for induction of remission in Crohn's disease [3].

Crohn's disease can cause patchy inflammation of the gastrointestinal system and affects the entire thickness of the bowel wall. Ulcerative colitis has continuous and uniform inflammation in the large intestine. Bloody stool is more common with ulcerative colitis and increased risk of cancer since inflammation persists longer and is associated with abnormal immune response, genetics, microbiome, and environmental factors, whereas Crohn's disease causes malnutrition. There is variation in the IBD depending on the climates and development status of the countries. In developed countries, there seem to be higher rates of IBD when compared to developing regions; similarly greater rates of IBD were identified in colder-climate regions and urban areas than those in warmer climates and rural areas [4]. Globally, the IBD incidence is around 0.1-16 cases per 100,000 person-years for Crohn's disease and 0.5-24.5 cases per 100,000 person-years for ulcerative colitis. Overall, the prevalence of IBD yearly is 396 cases per 100,000 persons. A review of IBD reported that the prevalence of Crohn's disease in Europe was 322 per 100,000 persons when compared to North America which was 319 per 100,000 persons. Prevalence rates for ulcerative colitis were 505 per 100,000 persons in Europe, whereas 249 per 100,000 persons in North America [5]. Both illnesses are characterized by the atypical response to the body’s natural defense. The burden of illness associated with CD is significant, with extended hospital stays and frequent emergency department visits, and more than 80% of patients with CD require hospitalization during their illness. In comparison with the normal population, health-related quality of life is significantly lower among CD patients [6-8].

Our study aimed to discuss the dosage and administration of ustekinumab and its challenges and importance in the treatment of IBD. We also outlined the mechanism of action and its safety and efficacy when compared to other drugs. Although there are many clinical trials, very little is understood about ustekinumab opportunities in the real world. Therefore this helps in understanding the role of antibodies and further research in comparison to biological treatment. The importance of our review is it summarizes the potential mechanism of action and treatment opportunities of ustekinumab compared to other biological treatments.

## Review

Methods

A Scopus review was performed and identified 465 relevant articles published in databases including PubMed, PubMed Central, CINHAL, Google Scholar, Scopus, Cochrane Library, and Web of Science. Additional publications were searched on Google Scholar from the reference lists of included studies and reviews by backward and forward snowball searches. A detailed literature search of the articles referenced in the identified publications was also performed within the last 10 years and subjects with age >18 years were included in the study. Articles describing ustekinumab and IBD, such as randomized control trials, clinical trials, original research articles, meta-analyses, and reviews, included 55 articles. Study protocol or study design, articles from non-English literature, abstracts, posters, commentaries, and studies on animals, children below 18 years of age, and pregnant women were excluded.

Results

Dosage, Administration, and Effectiveness

A multi-center study by Amiot et al. demonstrated both the efficacy and safety of ustekinumab in patients with ulcerative colitis. The cohort study consisted of patients who were refractory to other treatments, with recurrent drug failures. However, ustekinumab at weeks 12-16 demonstrated steroid-free clinical remission in as high as one-third of the cases [9].

With respect to dosing, a study conducted by Ollech et al. stated that a significant amount of patients with Crohn’s disease do not respond to the standard dose of ustekinumab of 90 mg every eight weeks. The study proceeded with performing a retrospective study to discover the effectiveness of ustekinumab dose interval shortening. They accomplished this by collecting data from 506 patients diagnosed with Crohn’s disease who had taken 90 mg ustekinumab subcutaneously every eight weeks. Along with the 506 participants, they also collected data from 110 patients with Crohn’s disease who had received ustekinumab subcutaneously also at 90 mg every eight weeks but then proceeded with their interval being shortened to every four weeks. The study revealed that those who were not responding to the dose every eight weeks indeed improved with changing the dosing interval to every four weeks. The improvement was seen in both the clinical aspect and the biological occurrence of the disease activity [10].

Another multicenter study conducted by Kopylov et al. focused on determining the effectiveness of dose escalation of ustekinumab. A total of 142 patients with Crohn's disease were included who had received a standard IV induction dose and at least one subcutaneous 90 mg dose of ustekinumab. The participants received a dose escalation by either shortening the interval to every four to six weeks, IV reinduction, or both. At week 16, 51.4% responded to the treatment, with 38.7% in clinical remission. On the last follow-up beyond week 16, 42% achieved clinical remission and 52% responded to the treatment. The study concluded with the idea that this strategy should be reviewed in cases of patients being unresponsive to the eight-week dosing treatment [11].

In a retrospective observational study by Haider et al., a total of 143 patients with Crohn’s disease who received ustekinumab over a 33-month period were included in the study. The study supported the four-week dose escalation for the patients refractory to the eight-week dosing treatment with ustekinumab. The study put forward that dose escalation not only improves clinical outcomes but also prevents the disease from worsening in severity. The study also claimed that dose escalation indeed helps improve the CRP and albumin levels in those with Crohn’s disease. The study suggests considering switching to the four-week dose escalation before suggesting a switch of medications to a different class of biological agents for the treatment of the disease [12].

Mechanism of Action 

Inflammatory bowel disease is an immune-mediated chronic inflammatory disease that is characterized by relapses and involves the gastrointestinal tract. The imbalance between innate and adaptive immune responses is the most important factor in disease severity which involves IL-12/IL-23 as a major trigger of inflammation in the human immune system [13]. The interleukin pathway plays an important role in the induction of inflammation. In particular, IL-23 promotes the differentiation of T-helper cells into Th17 with the secretion of inflammatory cytokines such as IL-17 and IL-22, whereas IL-12 induces the Th1 sand production of other cytokines, such as interferon-gamma and tumor necrosis factor.

Th1-type is induced by a microorganism that activates the secretion of interferon-gamma and IL-12p40 through the signal activation and transcription 1 (STAT1), T-box factor 21 (TBX21), and STAT4 cellular pathways. CD stimulates TH1-mediated pathology, which increases the synthesis of interferon (IFN)-gamma. In parallel, the inflamed intestinal mucosa is infiltrated by Th17 cells with the production of IL-17 cytokine. Th17 lineage is directed by transforming growth factor-beta (TGF-B) in the pro-inflammatory environment, and maintenance of Th17 cells. Moreover, IBD is characterized by increased production of IL-12, the major Th1 stimulating factor. The IL-12 family includes IL-12, IL-2, IL-35, and IL-27, which are considered key mediators of the inflammatory response [14]. The regulation of monocyte/macrophage differentiation, their survival, and functions in IBD are interesting in protein expression and inflammation of gastrointestinal mucosa. Interleukins are upregulated in lamina propria and isolated from the normal colon by tumor necrotic factor-alpha and toll-like receptor ligands [15].

**Figure 1:**
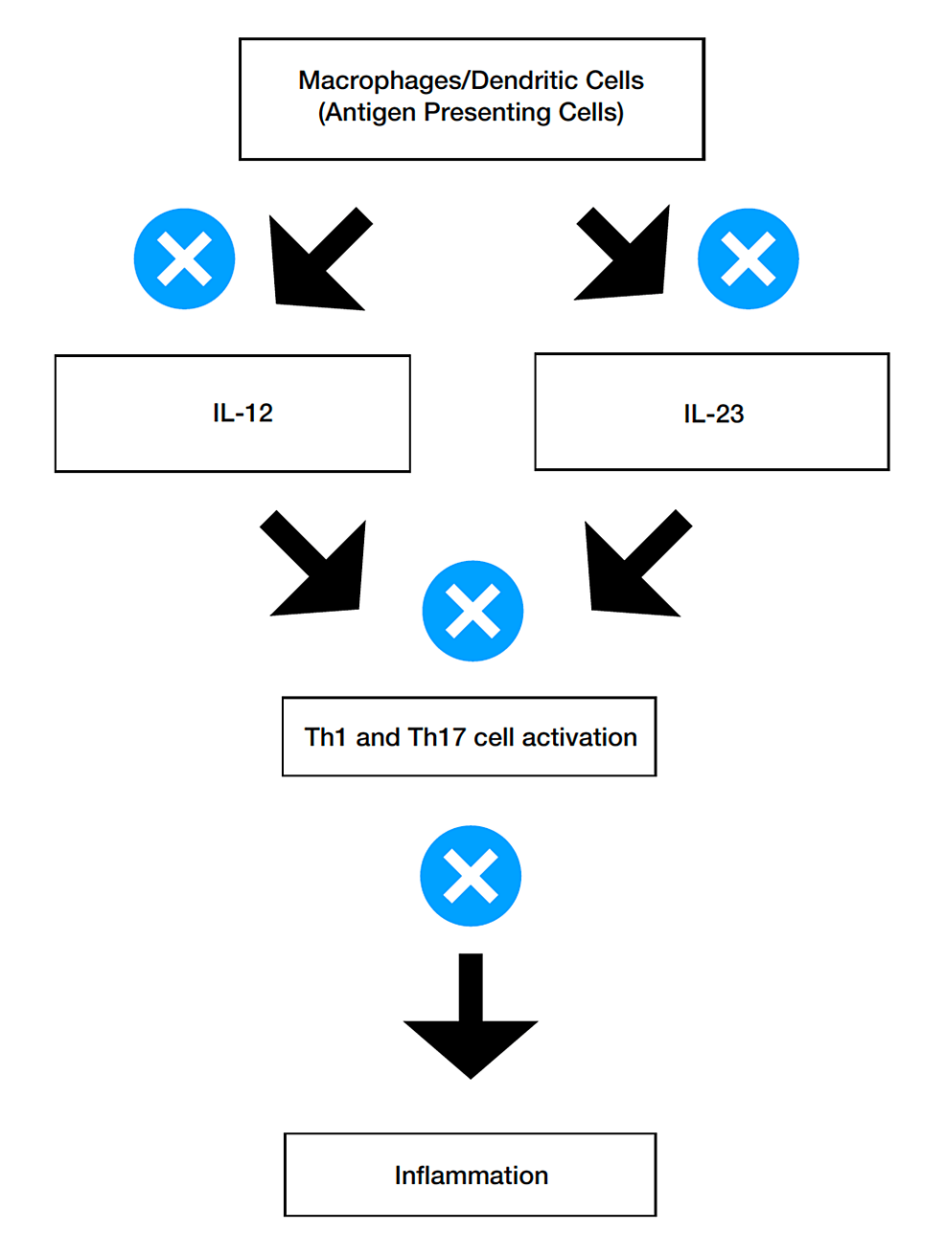
Ustekinumab - mechanism of action. X: inhibition (block/negative effect); IL: interleukin; Th: T helper cells

The interleukin 1 family consists of 11 proteins (IL-1F1-IL-1F11) which are encoded by 11 specific genes in humans. IL-1 type cytokines are certain mediators of immunity for most autoinflammatory diseases. IL-1 alpha and IL-2 beta, in turn, increase mRNA expression of hundreds of genes in different cell types [16]. Medical therapy in IBD is focused on non-specific immunosuppressive therapies including thiopurines and methotrexate. Ustekinumab is a human immunoglobulin G1 (IgG1) kappa monoclonal antibody that blocks IL-12 and IL-23 and has been approved for the treatment of moderate to severe IBD and psoriatic arthritis [17]. The results of phase II and phase III trials for ustekinumab treatment of IBD appear very encouraging. This has led to its approval by the Food and Drug Administration for the treatment of moderate to severe IBD in adults who have failed or were intolerant to treatment with immunomodulators or corticosteroids but never failed treatment with tumor necrotic factor (TNF) antagonists, or who failed or were intolerant to treatment with one or more tumor necrotic factor (TNF) blockers [18]. The mechanism of action is simplified as presented in Figure 1.

Effectiveness on Extraintestinal Manifestations

Extraintestinal symptoms (EIMs) are common in inflammatory bowel disease (IBD) patients and can be difficult to manage. Antibodies to tumor necrosis factor-alpha (anti-TNF) are widely accepted as the most effective treatment alternative. In a systematic review by Guillo et al., a total of 254 patients with IBD and EIM were enrolled in nine trials (eight retrospective and one prospective). Ustekinumab was shown to be successful in 152 patients with arthralgia and psoriatic arthritis in three high-quality trials. Seven studies proved its role in psoriasis, axial spondyloarthritis, pyoderma gangrenosum, and erythema nodosum. Ustekinumab has been shown to be an effective therapy for EIMs, especially in the cases of dermatological and rheumatological manifestations [19]. A retrospective chart review was performed on all patients with the diagnosis of CD who obtained ustekinumab (n=17) between January 2014 and December 2016. A total of 10 (63%) of 17 CD patients with a mean age of 38±3.21 were men, six (35%) of 17 were smokers, five (31%) of 16 patients had concurrent seronegative spondyloarthropathies, and three (18%) of 17 patients had dermatologic manifestations until treatment. Seronegative arthritis improved in two (40%) of five patients, mucosal healing was completed in four (40%) of 10 patients, and skin manifestations/psoriasis improved in two (66%) of three patients. Ustekinumab is an interleukin 12 receptor that has a strong steroid-free clinical induction and mucosal healing rate, as well as improved rheumatologic and dermatologic manifestations [20].

Matsumoto et al. reported a case of Crohn's disease with spondyloarthritis as an extraintestinal manifestation in which ustekinumab was found to be extremely successful in treating not only arthritis but also skin manifestations and scleritis [21]. A narrative review by Lambert et al. reveals the role of ustekinumab in dermatologic manifestations [22]. In around 5-20% of patients with inflammatory bowel disease, eczema may develop as a side effect of anti-TNF therapy (IBD). This possibility tends to be increased by a personal history of atopy. General precautions such as eliminating shower gel, using shower/bath oil, and adding emollients on a daily basis are advised. In recalcitrant situations, switching to ustekinumab therapy may be required. Psoriasiform lesions (paradoxical adverse event) were observed in 10.1% of 583 anti-TNF-treated IBD patients in a 14-year retrospective sample, which was mostly found in patients with CD (CD: 10.8% and UC: 6.8%). Anti-TNF-treatment along with other immunosuppressants has been associated with a lower risk of psoriasis. Since switching to ustekinumab, the majority of TNF-induced skin lesions have disappeared. In IBD patients with recalcitrant skin lesions, ustekinumab has been recommended as the medication of choice [23].

Discussion

Challenges and Opportunities in the Treatment With Ustekinumab in IBD Patients

Ustekinumab induces and maintains remission in moderate to severe luminal Crohn’s disease (CD) which is beneficial in disease course management. Those who had failed anti-TNFs in the pivotal unit I and unit II clinical trials also benefited from ustekinumab effectively. Ustekinumab was shown to be more effective than placebo in a phase II study of 526 patients refractory to anti-TNF. Patients received ustekinumab through intravenous induction followed by subcutaneous maintenance [24]. Different ustekinumab induction and maintenance regimens exist. However, patients with CD who received high doses subcutaneously showed superior results. Ustekinumab showed effectiveness in therapy-refractory or intolerant UC beyond clinical trials. A most recent phase III clinical trial (Understanding the Need for Improved Forest Intervention {UNIFI}) of ustekinumab of 961 UC patients demonstrates the effectiveness and safety of induction and maintaining remission. A related observational real-life study also confirms the result of the UNIFI, confirming ustekinumab to be an effective treatment option in patients with moderate to severe UC [25,26]. A 6 mg/kg body weight intravenous induction of ustekinumab, followed by a 90 mg subcutaneous injection once every eight weeks, is suggested. Patients are later evaluated at intervals of one, two, three, six, nine, and 12 months. Four of 16 patients with active disease were in clinical remission (clinical activity index {CAI} <4), a month after the start of a 12-month study (25%); eight patients at three, six, nine, and 12 months (50%) [27].

A recent multicenter retrospective cohort study was conducted to evaluate the efficacy of an IV dose re-induction of ustekinumab (6 mg/kg). A total number of 65 patients were recruited for either a partial response or secondary loss of response to ustekinumab. The study was based on biochemical, endoscopic, or clinical criteria. A total of 88.3% of patients were optimized once every four weeks prior to reinduction and clinical outcomes were analyzed at a median of 14 weeks (IQR: 12-19) post-reinduction. Clinical remission of corticosteroids with the biochemical and endoscopic response or remission was achieved in 31.0% of patients. Pre-reinduction ustekinumab concentrations were above 1 μg/mL in 88.6% of patients. No serious adverse event was reported following reinduction [28].

The association of serum concentrations of ustekinumab and various responses in patients with moderate to severe UC has been reported. The serum concentrations of ustekinumab were consistently found to be proportional to the dose among patients with UC. The outcomes also correlate with clinical and histologic efficacy and markers of inflammation. Serum concentrations of ustekinumab were dose-proportional and were at a steady-state concentration by the second maintenance dose among patients with UC. The median trough ustekinumab concentration was found to be threefold higher with every eight-week dosing compared to every 12-week dosing. A week eight post-induction concentration of 3.7 μg/mL was associated with response. A maintenance concentration of 1.3 μg/mL was correlated with increased rates of clinical remission. No adverse event was reported when administering higher serum concentrations of ustekinumab. Improvement in clinical features and normalization in histologic features were associated with serum concentration of the drug in ulcerative colitis patients [29].

Primary, partial, and loss of response are affected by disease-related elements that affect the pharmacokinetics and dynamics of biologics among patients. Reactive therapeutic drug monitoring (TDM) has become the standard of care for anti-TNF medications. A proactive TDM is increasingly performed even with conflicting data regarding its utility in clinical practice. The impact of measuring drug concentrations and anti-drug antibodies (ADA) levels for newer treatments, such as vedolizumab (VDZ), ustekinumab (UST), and tofacitinib, is not as clear as is for anti-TNF. An exposure-response relationship for these agents has been established in both CD and UC. Given the differences in action mode, drug pharmacokinetics, and immunogenicity compared to anti-TNF drugs, the value of TDM for treatment optimization with these agents is yet to be determined. Dose optimization is found to be efficient in regaining clinical response and remission in UST, VDZ, and tofacitinib [30].

Therapeutic drug monitoring of ustekinumab has improved IBD outcomes with higher drug durability; however, the clinical relevance of UST drug concentration and outcome is not quantitatively proven. The UST drug and anti-UST antibody concentration using three commercially available assays have shown a linear quantitative correlation. The Bland-Altman plots are also scattered and less linear. It is suggested that clinicians should use the same assay for a patient. The Kruskal-Wallis test confirmed a statistically significant difference between different assays of χ^2^(2)=30.606 (p<0.001) [31].

Using a sensitive drug-tolerant assay, the rates of antibodies to ustekinumab formation remained low through week 156; only 4.6% of all patients tested positive for antibodies at any point during maintenance. However, 8.2% of patients who were initially assigned to a placebo showed higher rates. Among, 2.4% of patients who remained on continuous once every eight weeks dosing without dose adjustment showed the lowest rates. Despite this, rates of antibody formation were similar among 4.5% of patients not receiving concomitant immunosuppressive compared to those on immunosuppressives at week 44 [32].

However, in a conflicting study, there seems to be a distinct exposure-response association with ustekinumab in the current literature. The exact threshold below which dose optimization may be useful needs to be determined across available commercial assays during clinical practice. Experts tend to agree on a target ustekinumab concentration of 3-7 μg/mL at week eight and 1-3 μg/mL during maintenance, at the minimum for both CD and UC [33].

Safety, Efficacy, and Clinical Outcomes of Ustekinumab in IBD Patients

Ustekinumab, a monoclonal antibody, is a useful treatment option in a patient with moderate to severe inflammatory bowel disease. Some data shows a reduction in anti-inflammatory markers in a patient after the use of ustekinumab. Without taking into consideration pharmacogenomics issues, the reported efficacy and safety data show that ustekinumab is a suitable treatment option for inflammatory bowel disease. In this study, we are going to look at the primary and secondary outcomes, efficacy, and safety data in the patients in the baseline before treatment and after a certain time of treatment [34,35].

In the study by Engel et al. in 2019, 578 patients were pooled for analysis. Most patients were treated with one of the biologicals [34]. At 52 weeks, the response rate was 49%, and the remission rate was 63% (95% CI: 0.53-0.72) approximately after one year. A total of 134 adverse events were reported on 19% of the patients among which 5% were serious and 6% of the patients reported infection. In the study by Thomas et al. in 2019, the outcome was studied in 19 patients. In five patients the treatment was held because of the side effects [27]. In 14 patients the median colitis index was decreased from 8.5 to 2.0 points in one year (range 1-12) and mayo endoscopy scores reduced from 2.0 points to 1.0 points at one year. In 53% of patients, clinical remission was achieved in one year [36,37].

In the study by Harris et al. in 2016, out of 45 patients, 46% achieved clinical response (Harvey-Bradshaw Index {HBI} decreases >3) and 35% achieved clinical remission (HBI decreases <3). ESR decreased from 20 (3-54) to 12 (0-42), p=0.005, and CRP decreased from 4.9 (0.3-11) to 3.3 (0.2-226) mg/dL, p<0.005. The endoscopic response was observed in 76% and 24% achieved complete endoscopic remission. Among 12 patients who were admitted for IBD-related issues, four experienced infection-related complications, six (13%) underwent surgery, three patients stopped using ustekinumab, and due to lack of response two and one stopped for a preference [38].

Regarding the safety issue, there was a consistent relationship between the dose of ustekinumab and infection or serious acute exacerbation. Regarding the efficacy variables, there was an inverse relation between ustekinumab concentration and CRP concentration. A positive association was observed between the concentration of the drug and positive outcomes in patients with Crohn's disease in both the maintenance phase and induction phase [39]. According to a study by Wils et al. in 2016, 79 patients showed clinical improvement after three months of treatment with ustekinumab. In the study by Wils et al., 93% and 68% of the patients showed clinical benefit after six months and 123 months after a median follow-up of 9.8 months [40]. Ustekinumab is also safe and does not increase the risk of recurrent cancer. It has a hazard ratio of 0.88 (with CI: 0.25-3.03). Patients with IBD and cancer history were exposed to anti-TNF inhibitors, vedolizumab and ustekinumab were tested and there was no increase in recurrent risk for cancer in either vedolizumab and ustekinumab after adjusting for age, IBD subtype, smoking, cancer stage. Patients with anti-TNF inhibitors and biological drugs were tested on recurrent or new cancers but there was an overall median of 5.2 person-years of follow-up [41].

Ustekinumab shows a rapid onset of clinical improvement with a high response rate within three weeks of treatment initiation. Its long-duration effect supports the fast onset efficacy shown by the results of the IM-UNITI long-term extension (LTE). A total of 75% of the patients achieved remission after one year [42]. The efficacy of the drug showed an advantage over anti-TNF agents. The efficacy of this drug is seen in those patients with adverse effects on frontline biologics who do not respond to other anti-TNF agents [43]. Similarly, in a study by Kopylov et al. in 2014, among 35 patients, 73.7% (28/38) achieved initial clinical response. Among the responders, 80% were able to maintain the response state for six months and also maintained the response at 12 months. At a follow-up of 7.9±5.2 months, 27 (71%) of 38 showed a good response rate and 73.3% were corticosteroid-free [44].

According to a study by Katherine et al. in 2020, there is a significant histo-endoscopic improvement and clinical and corticosteroid-free remission at 44 weeks [45]. According to Harris et al. in 2019, among 84 patients enrolled in the study, a post-inductive review of 72 patients and one year of data of 49 patients (53%) showed a clinical response and 8% of the patients had clinical remission in the post-induction phase. At one year, 71% of the patients showed a clinical response and 14% showed remission. Adverse effects were seen in four patients with infection requiring admission. Five patients underwent CD surgeries, two exacerbations, and one reported drug-related rash [46].

Thus from these studies, it can be shown that ustekinumab demonstrated good clinical response, long-term efficacy with some patients attaining remission, and improvement in biomarkers with very few adverse effects.

Cost Effectiveness

In patients who have failed traditional therapy, ustekinumab outperforms adalimumab. Hansson-Hedblom et al. studied cost-effectiveness in moderate to severe Crohn’s disease patients in Sweden and found out that ustekinumab is more cost-effective in the TNF-alpha-inhibitor failure population. Ustekinumab had a 94% chance of being more cost-effective than adalimumab and a 72% chance of being more cost-effective than vedolizumab [47]. Infliximab, adalimumab, and ustekinumab all generated comparable quality-adjusted life-years (3.5), but costs differed significantly ($50,510, $54,985, and $72,921 for infliximab, adalimumab, and ustekinumab, respectively) [48]. According to Holko et al., the cost-effectiveness of biologics is influenced by the use of ustekinumab/vedolizumab as a second-line anti-TNF [49]. Whereas in the study model of Sardesai et al., tofacitinib appears to be a cost-effective therapeutic choice for patients with mild to serious UC as compared to ustekinumab [50]. With the widespread use of less costly infliximab and adalimumab in the last decade, and now ustekinumab, rather than hospitalization or colectomy, biologics are now the primary driver of IBD management [51].

Comparison With Other Biological Treatments

Ustekinumab is a biological agent that has not only been shown to have the potential to provide benefits in cases of ulcerative colitis but also in cases of refractory treatment. Furthermore, a study by Biancone et al. in 2020 reported that this biological agent exhibits both effectiveness and safety in inducing and sustaining the treatment of ulcerative colitis [52]. According to a cohort study conducted by Kubesch et al. in 2019, ustekinumab was given to patients with Crohn’s disease, for which 96.2% of the patients were pre-exposed to anti-TNF agents. With the median follow-up of 49 weeks, 51.8% of the patients showed a response to ustekinumab, and 24.7% of patients were in remission. The study put forward that short- and long-term use of ustekinumab is both effective and tolerable [53]. Ustekinumab and tofacitinib with a surface under the cumulative ranking (SUCRA) score of 0.87 were ranked highest for induction of clinical remission. UST was superior to vedolizumab (OR: 5.99, 95% CI: 1.13-31.76) and adalimumab (OR: 10.71, 95% CI: 2.01-57.20) [54]. A review by Singh et al. in 2020 stated that in patients with prior exposure to TNF inhibitors, ustekinumab and tofacitinib were ranked highest for the induction of clinical remission. The study also propagated that these agents were superior to vedolizumab and adalimumab, other known biological agents [54]. In another cohort study conducted on ustekinumab therapy in infliximab-refractory pediatric ulcerative colitis by Dhaliwal et al. in 2021, ustekinumab showed efficacy in the treatment-refractory case demonstrating no adverse events with the therapy either [55].

## Conclusions

Ustekinumab can be considered an assuring treatment option in patients with Crohn's disease and ulcerative colitis. The drug plays a vital role in improving disease activity, endoscopic findings, and inflammatory markers. It shows high efficacy in patients with a lack of primary and secondary responses to TNF-alpha-blocking agents. It has a relatively better safety profile and is cost-effective as compared to other biological agents. Additionally, it is found to be extremely efficient in treating the extraintestinal manifestations of IBD. Ustekinumab can be used in older adults diagnosed with colorectal cancer. However, larger multicenter studies are essential in the future to learn in detail about the beneficial aspects of ustekinumab in treating patients with Crohn’s disease and ulcerative colitis.
